# Supplementary material for: Formulation and Characterization of Mucoadhesive Polymeric Films Containing Extracts of Taraxaci Folium and Matricariae Flos
Source: Molecules. 2023 May 10;28(10):4002. doi: 10.3390/molecules28104002 (PMC10220881; doi:10.3390/molecules28104002)
Supplement: Supplementary file 1 [file molecules-28-04002-s001.zip › molecules-2352210-supplementary.pdf]

1. Statistical analysis for Tabel S1 and Tabel S3 of the manuscript

Tabel S1. TPC statistical analysis

M vs. T	0,0002	***
M vs. T/M 1:1	<0.0001	****
M vs. T/M 1:2	0,0004	***
M vs. T/M 2:1	0,0002	***
T vs. T/M 1:1	0,9645	ns
T vs. T/M 1:2	0,9645	ns
T vs. T/M 2:1	>0.9999	ns
T/M 1:1 vs. T/M 1:2	0,707	ns
T/M 1:1 vs. T/M 2:1	0,9338	ns
T/M 1:2 vs. T/M 2:1	0,9841	ns

*statistically significant differences for p<0.05

Tabel S2. TFC statistical analysis

**Tukey's multiple comparisons test**	**Adjusted P Value**	**Summary**
M vs. T	<0.0001	****
M vs. T/M 1:1	<0.0001	****
M vs. T/M 1:2	<0.0001	****
M vs. T/M 2:1	<0.0001	****
T vs. T/M 1:1	<0.0001	****
T vs. T/M 1:2	<0.0001	****
T vs. T/M 2:1	<0.0001	****
T/M 1:1 vs. T/M 1:2	<0.0001	****
T/M 1:1 vs. T/M 2:1	0,0026	**
T/M 1:2 vs. T/M 2:1	<0.0001	****

*statistically significant differences for p<0.05

Tabel S3. DPPH statistical analysis

**Tukey's multiple comparisons test**	**Adjusted P Value**	**Summary**
M vs. T	<0.0001	****
M vs. T/M 1:1	<0.0001	****
M vs. T/M 1:2	0,0035	**
M vs. T/M 2:1	<0.0001	****
T vs. T/M 1:1	0,0173	*
T vs. T/M 1:2	<0.0001	****
T vs. T/M 2:1	0,0707	ns
T/M 1:1 vs. T/M 1:2	0,0176	*
T/M 1:1 vs. T/M 2:1	0,8864	ns
T/M 1:2 vs. T/M 2:1	0,0045	**

*statistically significant differences for p<0.05

Tabel S4. Cuprac statistical analysis

Tukey's multiple comparisons test	Adjusted P Value	Summary
M vs. T	<0.0001	****
M vs. T/M 1:1	<0.0001	****
M vs. T/M 1:2	<0.0001	****
M vs. T/M 2:1	<0.0001	****
T vs. T/M 1:1	<0.0001	****
T vs. T/M 1:2	<0.0001	****
T vs. T/M 2:1	<0.0001	****
T/M 1:1 vs. T/M 1:2	0,0001	***
T/M 1:1 vs. T/M 2:1	<0.0001	****
T/M 1:2 vs. T/M 2:1	0,0215	*

*statistically significant differences for p<0.05

2. The HPLC chromatograms

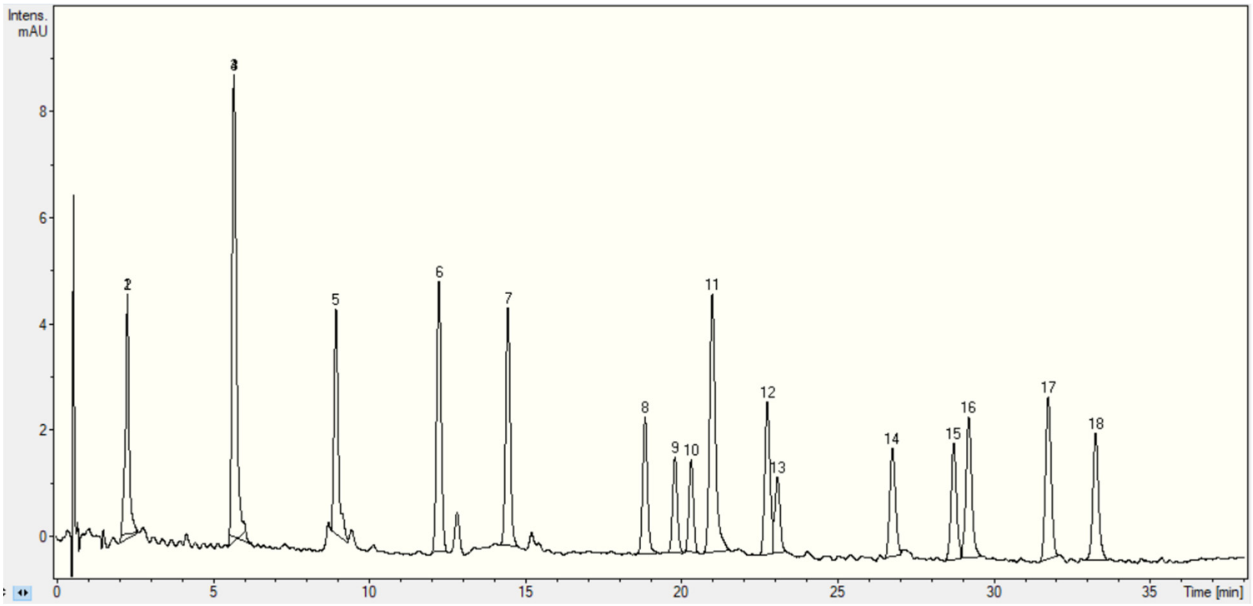

Figure S1. Standard sample HPLC chromatogram

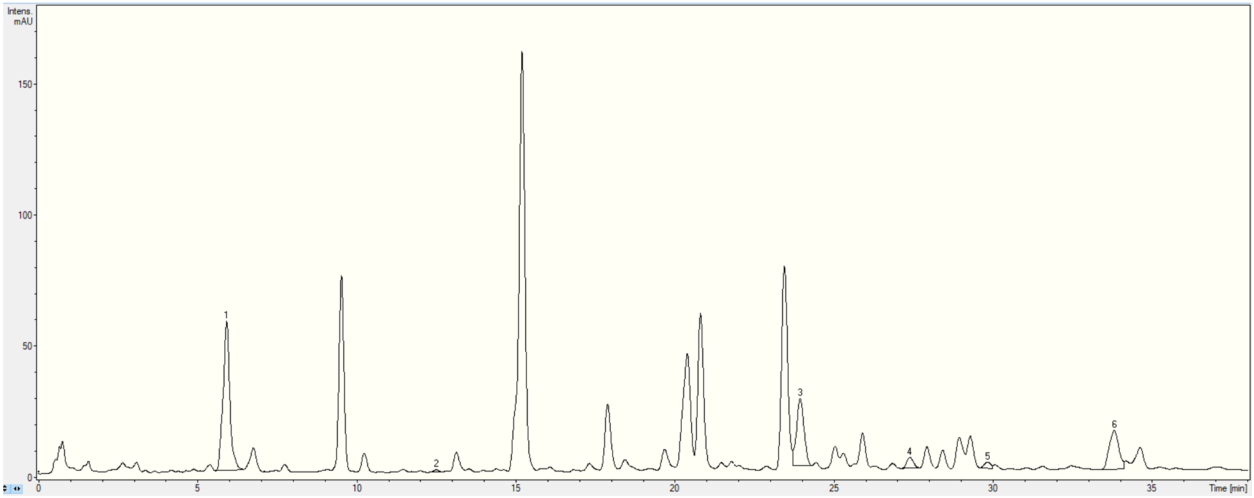

Figure S2. Matricariae flos sample HPLC chromatogram

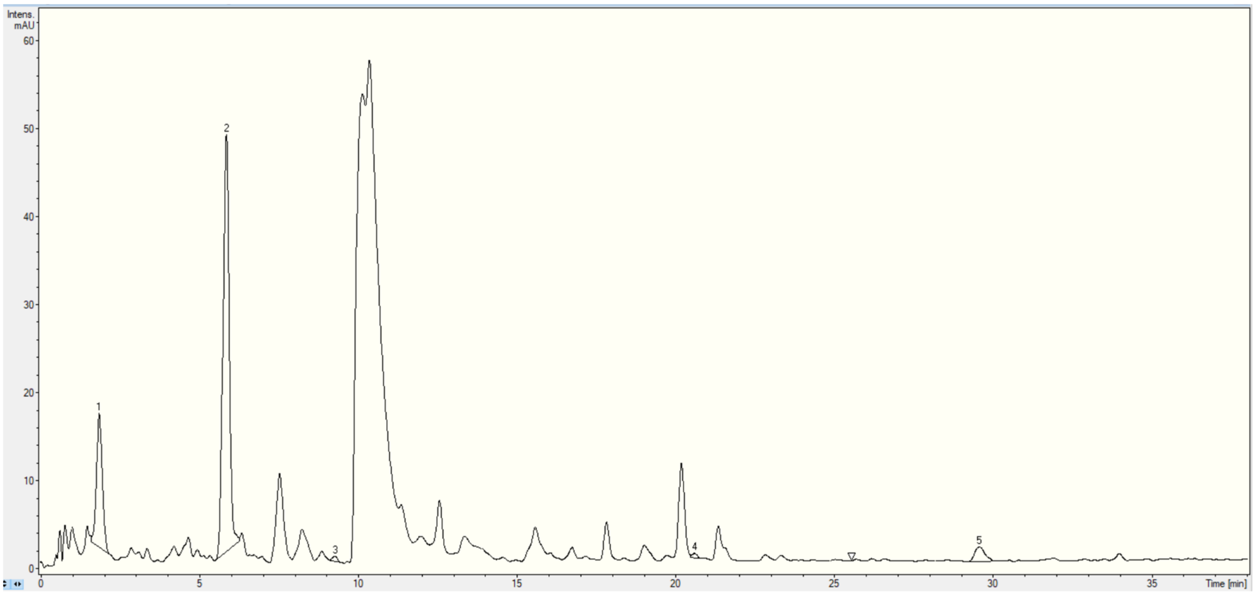

Figure S3. Taraxaci folium sample HPLC chromatogram
